# Alleviating effects of pea peptide on oxidative stress injury induced by lead in PC12 cells *via* Keap1/Nrf2/TXNIP signaling pathway

**DOI:** 10.3389/fnut.2022.964938

**Published:** 2022-08-11

**Authors:** Ning Li, Liuding Wen, Fangyu Wang, Tiange Li, Haodan Zheng, Tianlin Wang, Mingwu Qiao, Xianqing Huang, Lianjun Song, Erkigul Bukyei, Mingming Li

**Affiliations:** ^1^College of Food Science and Technology, Henan Agricultural University, Zhengzhou, China; ^2^Key Laboratory for Animal Immunology, Henan Academy of Agricultural Sciences, Zhengzhou, China; ^3^Department for Food Engineering and Hydromechanics, School of Engineering and Technology, Mongolian State University of Life Sciences, Ulaanbaatar, Mongolia

**Keywords:** lead, Keap1, Nrf2, TXNIP, oxidative stress, pea peptide, PC12 cell

## Abstract

**Background:**

Lead poisoning causes an oxidative stress response – a key “bridge” connecting various pathways – in the human body. Oxidative stress usually implies an imbalance between pro-oxidants and antioxidants. Moreover, Nrf2, Keap1, and TXNIP proteins play an essential role in oxidative stress. Some studies showed that pea peptides could alleviate the oxidative stress response. However, the effect and mechanism of pea peptide on oxidative stress response induced by lead in PC12 cells has not been reported.

**Aim:**

Investigating the effect and mechanism of pea peptides in alleviating oxidative damage in PC12 cells induced by lead.

**Methods:**

In this study, cell viability was measured by CCK8 (Cell Counting Kit-8). Superoxide dismutase (SOD), catalase (CAT), glutathione (GSH), glutathione reductase (GR), glutathione peroxidase (GPx), reactive oxygen species (ROS), and lipid peroxidation (MDA) were measured using the corresponding Biochemical kits. The Keap1, Nrf2, and TXNIP protein expressions were tested using Western blot.

**Results:**

Pea peptides PP3, PP4, and PP6 could reverse the decrease of cell viability caused by lead exposure (*P* < 0.05), the elevation of ROS and MDA caused by lead exposure, and the decrease of CAT, SOD, GR, GPx, and GSH/GSSG caused by lead exposure (*P* < 0.05). Moreover, PP3, PP4, and PP6 could reduce the elevated expression of Keap1 and TXNIP caused by lead exposure; and increase the expression of Nrf2 (*P* < 0.05).

**Conclusion:**

PP3, PP4, and PP6 can alleviate lead-induced oxidative stress damage in PC12 cells, and the Nrf2/Keap1/TXNIP signaling pathway may play an essential role in this process.

## GRAPHICAL ABSTRACT

This figure illustrates the mechanism of action of PP3, PP4, and PP6 in mitigating oxidative stress due to lead exposure. When PC12 cells are exposed to lead, large amounts of ROS are produced, leading to oxidative stress. (1) The intracellular antioxidant system is inhibited, and large lipid peroxides are produced. (2) The intracellular expression level of Keap1 is increasedand the expression level of Nrf2 is decreased and translocated to the nucleus by dissociating from Keap1 in the cytoplasm. (3) The intracellular level of TXNIP is increased and translocated from the nucleus to translocate to the cytoplasm and mitochondria to bind to TRX and inhibit the antioxidant system of the organism. (4) Pre-incubation with PP3, PP4 and PP6 reversed this phenomenon. 
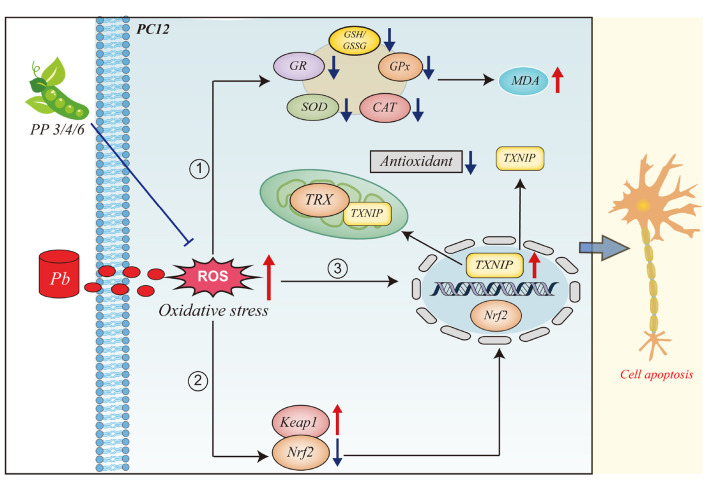

## Introduction

According to the World Health Organization, lead poisoning remains a major environmental health threat and a continuing source of health disparities. It has been defined as one of the ten chemicals addressing major public health issues. It is well known that lead exposure during early childhood can damage the central nervous system and lead to various cognitive problems such as mental disorders, attention deficit and hyperactivity disorder (ADHD), and low IQ (1). In Asia, the Middle East, and elsewhere, lead levels in seafood and meat products usually exceed the standard set by the European Commission. Lead can enter and accumulate in the body through the digestive tract, respiratory tract and skin contact. When it accumulates to a certain level, it can lead to lead poisoning (2).

Lead poisoning causes oxidative stress in the body, which has been reported as one of its potential mechanisms (3). Oxidative stress is an imbalanced state between oxidative and antioxidant actions in the body. It promotes oxidation process and leads to inflammatory infiltration of neutrophils and increased secretion of proteases, resulting in the production of large amounts of oxidative intermediates (4). The antioxidant system in the body consists of antioxidant enzymes and non-enzymatic antioxidants. The antioxidant enzymes include superoxide dismutase (SOD), catalase (CAT), glutathione reductase (GR), and glutathione peroxidase (GPx). Non-enzymatic antioxidants consist of vitamin C, vitamin E, and carotenoids (5). Many epidemiological and experimental studies demonstrated that lead could induce oxidative stress. Oxidative stress caused by lead exposure may lead to the development of hypertension and cardiovascular disease (6). Meanwhile, it could also affect the hematological system through oxidative stress-induced erythrocyte damage (7).

The NRF2-KEAP1 signaling pathway is a significant regulator of the antioxidant response to oxidative stress. Lead exposure aggravates oxidative stress by activating the Nrf2/Keap1 pathway (2). Kelch-like ECH-associated protein 1 (Keap1) is an essential chaperone for E3 ubiquitin ligase, and Nrf2 signaling plays a crucial role in regulating the cellular defense and anti-inflammatory responses to oxidative stress (8). Thioredoxin-interacting protein (TXNIP) is endogenous thioredoxin (TRX) repressor protein widely expressed *in vivo*. In cells, thioredoxin-interacting protein is the only protein in the α-arrestin family that can bind to TRX. Cysteine 63 and cysteine 247 in TXNIP form disulfide bonds with the hydrophobic group of the active site of reduced TRX, thereby inhibiting the antioxidant function of TRX and contributing to ROS accumulation (9). It has been found that the effect of TXNIP on TRX/ROS is blocked by the p38 MAPK signaling pathway inhibitor SB03580, suggesting that TXNIP is involved in the regulation of intracellular ROS by p38 MAPK (10).

Proteins extracted from pea have several biological functions. For example, the sticky soluble polysaccharides in peas can improve glucose tolerance, reduce blood lipid, and increase the secretion of ileal bile acid (11). Pea protein reduces blood glucose in streptozotocin (STZ) induced diabetic mice effectively (12) and hydrolyzate can lower blood pressure in hypertensive rats (13). Moreover, pea seed protein showed anti-inflammatory effects in a mouse model of colitis (14). It was reported that the protein isolated from mung beans exhibited several antioxidant activities (15).

However, the alleviating effects of pea peptides on oxidative stress injury induced by lead in PC12 cells have not been explored clearly. Therefore, this study aimed to (1) investigate whether pea peptides could alleviate the oxidative stress damage caused by lead in PC12 and (2) the role of the Keap1/Nrf2/TXNIP signaling pathway in this process.

## Materials and methods

### Materials

High-glucose Dulbecco’s modified Eagle’s medium (DMEM), fetal bovine serum (FBS), ROS assay kits, superoxide dismutase activity (SOD) assay kits, reduced and oxidized glutathione (GSH and GSSG) assay kit, and malondialdehyde (MDA) assay kit were purchased from Solarbio (Beijing, China). The activities of catalase (CAT), glutathione reductase (GR), and glutathione peroxidase (GPx) were detected using the corresponding test kits (Beyotime Biotechnology, Nanjing, China). Primary antibodies against β-actin, Keap1, TXNIP, and Nrf2 were purchased from Solarbio (Beijing, China). Conjugated anti-rabbit and anti-mouse antibodies were purchased from Proteintech (Wuhan, China). Unless otherwise stated, all other chemicals were of analytical grade and purchased from Solarbio (Beijing, China) and Beyotime (Shanghai, China).

Table 1 shows the sequences and related information of the six pea peptides. All peptides used in this study were synthesized by GL Biochem Ltd. (Shanghai, China), and their purity was greater than 80%.

**TABLE 1:** The biological activity, water solubility, toxicity and their functions of polypeptides.

NO.	Sequence	Functions	Static charge	Activity score	Toxicology	Water soluble
PP1	EFEGMTFLL	Anti-tumor	−2	0.639	Non-Toxin	Poor
PP2	KGQTPLFPR	Antioxidant	+1	0.718	Non-Toxin	Good
PP3	KYSSPIHIW	Antibacterial, Antioxidant	+1.1	0.626	Non-Toxin	Poor
PP4	KKADLYNPR	Antibacterial, Antioxidant	+2	0.466	Non-Toxin	Good
PP5	EHYDSEAILF	Antibacterial, Antioxidant	−2.9	0.407	Non-Toxin	Good
PP6	KYGPTPVRDGFK	Antioxidant	+2	0.451	Non-Toxin	Good

### Cell culture and treatment

Rat pheochromocytoma cell line PC12 cells were obtained from the Animal Immunology Laboratory, Henan Provincial Academy of Agricultural Sciences (Zhengzhou, China). The cells were cultured in Dulbecco’s Modified Eagle’s Medium (DMEM), supplemented with 10% (v/v) heat-inactivated fetal bovine serum (FBS) at 37°C in a 5% CO_2_ incubator. Logarithmic growth phase cells were taken for subsequent experiments.

To determine whether pea peptides can have a protective effect against lead injury, this study followed the protocol of Cheng et al. (16) and used pea peptides preincubated for 4 h prior to lead exposure. First, PC12 cells at the logarithmic growth stage were pre-incubated in 6-well plates for 24 h. Then the medium was replaced with fresh medium with or without peptides for 4 h. Finally, the medium was replaced with fresh medium with or without lead for 24 h for subsequent assays. This experiment contains six groups: the control group, Pb group, PP3 + Pb group, PP4 + Pb group, PP6 + Pb group and Vc + Pb group.

### Measurement of reactive oxygen species level

Reactive oxygen species level was measured according to the method of Li et al. (17). Briefly, DCFH-DA was used to co-incubate the cells with different treatments for 30 min. The cells were then washed off without residual DCFH-DA using PBS. The level of ROS was detected using the Multi-Mode Detection Platform (Spectar Max i3) with an excitation wavelength of 488 nm and an emission wavelength of 525 nm.

### Measurement of malondialdehyde level

Malondialdehyde was measured by the lipid preoxidation MDA kit. In short, the lysed cells were reacted with thiobarbituric acid at 100°C for 15 min. MDA levels were then detected using a Multi-Mode Detection Platform (Spectar Max i3) at 532 nm. MDA was calculated using a standard curve according to the manufacturer’s data sheet.

### Measurement of GSSG and glutathione level

Glutathione and GSSG was measured according to the method of Xu et al. (18). The whole process contains following steps. First, this experiment produced total glutathione content by reacting the sample with the assay working solution at 25°C for 5 min. After this, NDAPH was added and the absorbance value was detected at 412 nm every 5 min using a microplate reader and maintaining the temperature at 25°C. For the GSSG assay, the sample was reacted with GSH scavenging reagent at 25°C for 60 min, and then proceeded as above.

### Measurement of antioxidant enzymes

#### Measurement of catalase activity

Catalase activity was measured according to the method of Zhao et al. (19). Briefly, the lysed cells were mixed with phosphate buffer and hydrogen peroxide. Detection was performed in kinetic form using the Multi-Mode Detection Platform (Spectar Max i3) with an absorbance of 240 nm. The CAT activity was calculated according to the standard curve provided by the reagent vendor.

#### Measurement of superoxide dismutase activity

Total SOD activity was measured according to the method of Chen et al. (20). In short, the collected samples were mixed with the SOD assay working solution and then added to the starter working solution. After incubation at 37°C for 30 min, the SOD activity was measured using Multi-Mode Detection Platform (Spectar Max i3) with an absorbance of 450 nm. SOD activity was calculated according to the formula provided by the reagent vendor.

#### Measurement of glutathione reductase and glutathione peroxidase activity

The intracellular GR and GPx levels were detected separately according to the method of Guo and Zhang et al. (21, 22). In short, for GR, the sample was mixed with GSSG solution and NADPH solution, DTNB solution was added, and the absorbance value at 412 nm was detected using a microplate reader and kept at 25°C. For GPx, the sample was mixed with GPx assay working solution and hydrogen peroxide reagent solution, and the absorbance value at 340 nm was detected using a microplate reader at 25°C.

### Expression of Keap1/Nrf2/TXNIP pathway proteins

PC12 cells were processed according to different groups and the method to Western blotting analysis follows procedures used in Rahman et al. (23). In a nutshell, the PC12 cells were washed 3 times with pre-chilled PBS. The 200 μl of RIPA lysate containing protease inhibitor was added to each well. All wells were shaken on ice for 30 min, and collected and centrifuged at 12,000 rpm for 15 min, and finally, the concentration of the collected protein supernatant was assayed with the BCA protein concentration assay kit.

Total protein (40 μg) of each sample was separated by electrophoresis using 10% sodium dodecyl sulfate-polyacrylamide (SDS-PAGE) and then transferred onto polyvinylidene fluoride (PVDF) membrane using the wet transfer method. Afterward, the membranes were closed for 2 h at room temperature using 5% skim milk. Next, the membranes were incubated with the desired primary antibody overnight at 4°C in a shaker. The membranes were incubated with the corresponding secondary antibody with horseradish peroxidase at room temperature for 1 h. Finally, the proteins on the PVDF membranes were visualized using ECL luminescence reagents. The images of the detected bands were analyzed using Fusion FX6-XT, and the density of the protein bands was analyzed using Image-Pro Plus 6.0 software. The intensities of the bands were compared to that of β-actin (internal control). To ensure reproducibility, experiments were performed at least in triplicate.

### Statistical analysis

All presented data are the mean results of at least three independent measurements. Data are expressed as mean ± standard deviation (SD) of the independent experiments. When it was necessary to compare three or more groups of data, a one-way ANOVA and LSD test was used. Statistical analysis of the data was performed using the SPSS 20 statistical package (IBM Corporation, Armonk, NY, United States). The statistical results were considered statistically significant when *P* < 0.05.

## Results and discussion

### Preventive effects of pea peptides on cell viability

**FIGURE 1:**
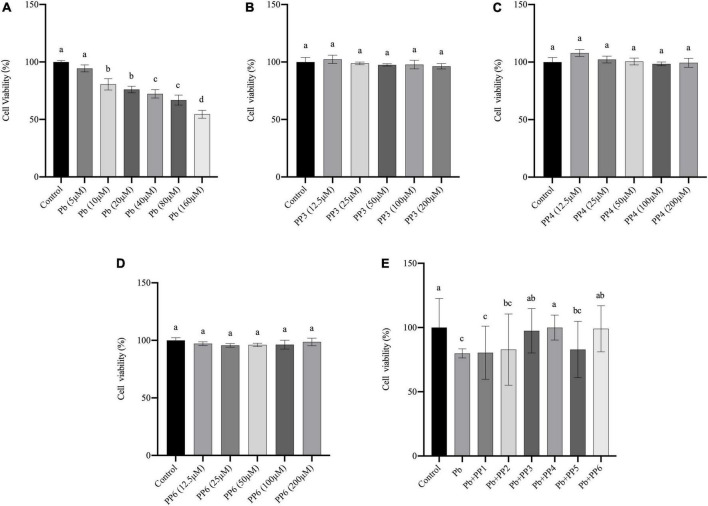
Effect of different treatments on cell viability of PC12 cells. **(A)** Effects of lead on cell viability in PC12 cells. PC12 cells incubated with lead (0–160 μM) for 24 h. **(B–D)** Effects of PP3, PP4, and PP6 cell viability in PC12 cells. PC12 cells were incubated with **(B)** PP3, **(C)** PP4, and **(D)** PP6 for 4h. **(E)** Effects of PP1-6 on cell viability in lead-exposure PC12 cells. PC12 cells were pre-incubated with PP1-6 (200 μM) for 4 h, then treated with 10 μM lead for 24 h. The valued of the bars indicates the means ± SD (*n* = at least 3). Different superscript letters indicate differences (*P* < 0.05).

Lead exposure is known to cause oxidative stress, leading to apoptosis or necrosis of PC12 cells (24). In our preliminary experiments, the damage of lead on PC12 cells was first assessed. CCK-8 results showed that 10 μM of lead caused a significant decrease (*P* < 0.05) in cell viability compared to the control group, indicating that lead caused severe damage to PC12 cells at this concentration. Lead at 40 μM caused a further decrease (*P* < 0.05) in cell viability compared to lead at 10 μM. Cell viability was further reduced when the concentration of lead is 160 μM (*P* < 0.05). It indicated that lead exposure leads to a dose-dependent decrease in cell viability (Figure 1A). Therefore, the lowest damage concentration of 10 μM was selected for the next steps of the experiment.

To determine whether pea peptide causes harm to PC12 cells, this study used cells incubated for 4 h with different concentrations of pea peptide to observe the changes in cell viability. The results from Figures 1B–D showed that 12.5–200 μM pea peptide did not cause damage to the cells (*P* < 0.05). Therefore, 200 μM pea peptide was selected for the next experiment in this study. Besides, this study pre-incubated the pea peptide for 4 h and then incubated with lead for 24 h to test whether the pea peptide had a protective effect on the lead-exposed cells. As shown in Figure 1E, pre-incubation with PP3, PP4, and PP6 before lead treatment effectively increased cell viability and restored it to the control level (*P* < 0.05). Other studies have reported that enzymatic protein hydrolyzates from yellow pea seeds can enhance the reduction in cell viability caused by high sugar, which could be an effect caused by their rich biological activity (25).

### Effects of pea peptides on reactive oxygen species level

Reactive oxygen species have an essential role in lead-induced neurotoxicity, and their elevated levels indicate that cells are subjected to oxidative stress. Ascorbic acid (VC) has a powerful antioxidant effect. Based on the relevant literature, this study chose 80 μM of VC as the standard for the control group to evaluate the antioxidant properties of pea peptides (26).

**FIGURE 2:**
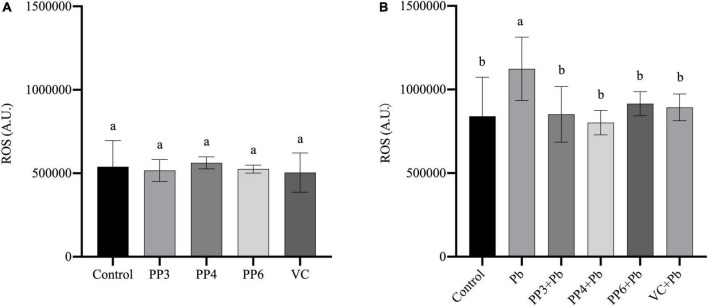
Effects of PP3, PP4, PP6 and VC on intracellular ROS in PC12 cells. **(A)** PC12 cells incubated with PP3, PP4, PP6, and VC for 4 h. **(B)** PC12 cells pre-incubated with PP3, PP4, PP6, and VC for 4 h, then treated with lead for 24 h. The valued of the bars indicates the means ± SD (*n* = at least 3). Different superscript letters indicate differences (*P* < 0.05).

This study examined the effects of three pea peptides on reactive oxygen species in PC12 cells (Figure 2A). This study found that 200 μM of PP3, PP4, and PP6 did not increase intracellular ROS levels compared to the control, and the results of VC were consistent with them (*P* < 0.05). Then, this study examined the effect of pretreatment with pea peptides for 4 h prior to lead exposure on ROS. As Figure 2B shows, the lead treatment significantly increased ROS in the cells compared to the control group. PP3, PP4, PP6, and VC preincubation significantly decreased ROS generated by lead exposure compared to the lead group (*P* < 0.05). In one study on the treatment of arsenic poisoned mice, ROS levels in the uterus of mice treated with pea protein were significantly reduced, demonstrating that it inhibited oxidative stress caused by arsenic poisoning (27). This study also illustrated that hydrogen peroxide and lipopolysaccharide-induced ROS in RAW264.7 cells increased significantly and that treatment with wood pea extract reversed this result (28).

### Effects of pea peptides on antioxidant system

Several enzymes, including SOD, CAT, GR, and GPx, play a crucial role in scavenging reactive oxygen species and reducing oxidative damage in the antioxidant system. GPx refers to an enzyme that uses GSH as a reaction substrate to reduce hydrogen peroxide to water or the corresponding alcohols. SOD, CAT, and GPx form an essential anti-redox system in the organism, preventing the cell membrane and other biological tissue functions from being disrupted by oxidative stress (29). GR is one of the essential enzymes in the human redox system. It is the significant flavins that maintain the prototype GSH content in cells. Glutathione is a tripeptide bound by glutamate, cysteine, and glycine, with antioxidant effects and integrative detoxification. Also, the production of lipid oxidation products is one of the main events of oxidative cell damage and can be assessed by the MDA level.

**FIGURE 3:**
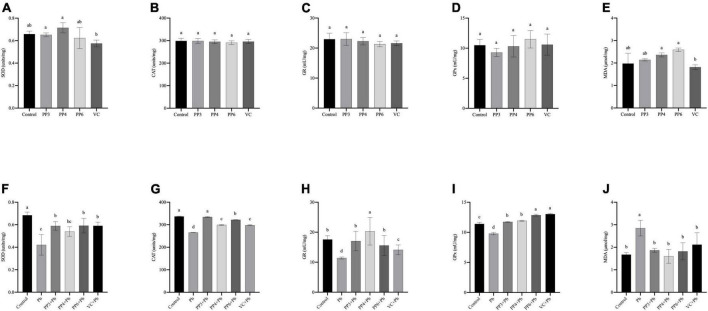
Effect of PP3, PP4, PP6, and VC on Oxidative stress indicators in PC12 cells. **(A–E)** PC12 cells incubated with PP3, PP4, PP6, and VC for 4 h. **(F–J)** PC12 cells were pre-incubated with PP3, PP4, PP6, and VC for 4 h, then treated with lead for 24 h. The results of T-SOD are shown in panels **(A,F)**. The results of CAT are shown in panels **(B,G)**. The results of GR are shown in panels **(C,H)**. The results of GPx are shown in panels **(D,I)**. The results of MDA are shown in panels **(E,J)**. The valued of the bars indicates the means ± SD (*n* = at least 3). Different superscript letters indicate differences (*P* < 0.05).

This study examined the effects of three pea peptides and VC on SOD, CAT, GR, GPx and MDA of PC12 cells (Figures 3A–E). Compared with the control group, the three pea peptides and VC did not cause significant effects on these antioxidant enzymes and MDA in PC12 cells (*P* < 0.05), indicating that the pea peptides and VC did not cause oxidative damage to PC12 cells.

The effect of preincubation with three pea peptides and VC before lead treatment on the above indices was further tested (Figures 3F–J). Compared with the control group, the lead treatment caused a significant decrease in the activities of SOD, CAT, GR, and GPx (*P* < 0.05). It increased the MDA content, indicating that lead treatment led to oxidative damage in PC12 cells. Lead treatment was reported to cause oxidative damage to PC12 cells, It decreases the activity of antioxidant enzymes and increases the production of lipid peroxides (30). PP3, PP6, and VC significantly increased the SOD activity compared to the lead group, but PP4 did not (Figure 3F); however, none of these pretreatments restored SOD activity to normal levels (*P* < 0.05). Compared with the lead group, PP3, PP4, PP6, and VC all significantly increased CAT activity, with PP3 having a better effect than PP6 and PP6 having a better effect than PP4 and VC (Figure 3G); nonetheless, only PP3 returned to the normal level (*P* < 0.05). The results of the GR assay are shown in Figure 3H. Compared with the lead group, PP3, PP4, PP6, and VC all significantly increased GR viability; compared with the control group, PP4 significantly increased GR viability, and both PP3 and PP6 restored GR viability to the level of the control group (*P* < 0.05). As shown in Figure 3I, PP3, PP4, PP6, and VC significantly increased GPx activity compared to the lead group; interestingly, GPx levels in all the experimental groups were significantly increased compared to the control group, and the effect of VC and PP6 was better than that of PP3 and PP4 (*P* < 0.05). The results of the MDA assay are shown in Figure 3J. Compared with the lead group, PP3, PP4, PP6, and VC significantly reduced the rise in MDA caused by lead: the corresponding date in all groups returned to normal levels (*P* < 0.05).

Many bioactive peptides isolated from plants have antioxidant activity. For example, peptides isolated and identified from wheat, lupine, and peas can effectively scavenge superoxide anions, hydroxyl radicals and inhibit lipid peroxidation (31). Pea protein hydrolyzates prepared with different proteases demonstrated excellent antioxidant activity under a liposome model system (32). Pea peptides have also been reported to reduce oxidative stress caused by lead exposure and be involved in reducing and scavenging excess ROS in cells (33).

### Effects of pea peptides on glutathione/GSSG

**FIGURE 4:**
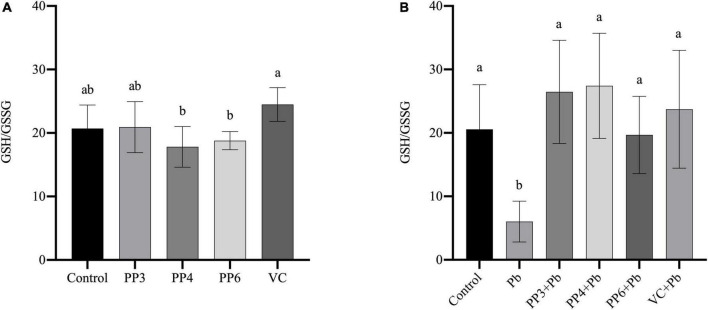
Effect of PP3, PP4, PP6, and VC on GSH/GSSG in PC12 cells. **(A)** PC12 cells were incubated with PP3, PP4, PP6, and VC for 4 h. **(B)** PC12 cells were pre-incubated with PP3, PP4, PP6, and VC for 4 h, then treated with lead for 24 h. The valued of the bars indicates the means ± SD (*n* = at least 3). Different superscript letters indicate differences (*P* < 0.05).

The redox status of cells can be reflected by the ratio of GSH/GSSG. This study examined the effects of pea peptides and VC on GSH/GSSG in PC12 cells (Figure 4A). The three pea peptides and VC did not significantly decrease GSH/GSSG (*P* < 0.05), demonstrating that they did not affect the normal glutathione redox cycle in PC12 cells. Then, the effects of pea peptides and VC preincubation on GSH/GSSG were further examined (Figure 4B). In this study, lead treatment could significantly decrease the level of GSH/GSSG, demonstrating its effect on the glutathione redox cycle. Lead treatment has been shown to decrease intracellular GSH levels, which is consistent with our findings (34). When preincubated with pea peptides and VC for lead treatment, PP3, PP4, PP6, and VC significantly increased GSH/GSSG and returned to normal levels compared to the lead group (*P* < 0.05). It has been reported that tea catechins can dramatically reverse the decrease in GSH/GSSG ratio caused by lead exposure (35). Cytidylcholine significantly increased the reduced glutathione in PC12 cells to antagonize the oxidative stress produced by lead exposure (36).

### The influence of pea peptides on Keap1/Nrf2/TXNIP signal pathway

Nuclear factor E2-related factor 2 (Nrf2) is an integral part of the cellular self-defense system against exogenous stimuli and is sensitive to oxidative stress. When the organism is exposed to oxidative stress, Nrf2 dissociates from Keap1 in the cytosol and enters the nucleus. When the organism produces excessive ROS, TXNIP enters the cytoplasm and mitochondria from the nucleus to bind with thioredoxin (TRX), which inhibits the antioxidant capacity of TRX and further leads to the accumulation of ROS (37).

**FIGURE 5:**
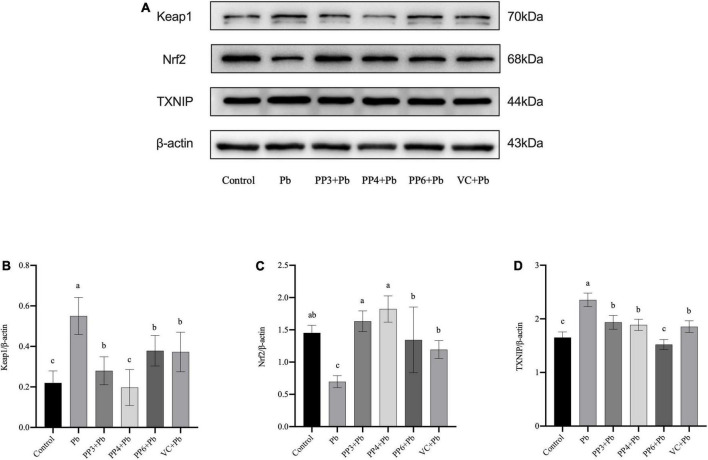
Western blotting analysis of the effect of PP3, PP4, PP6, and VC on Keap1, Nrf2, and TXNIP expression in lead-exposed PC12 cells. PC12 cells were pre-incubated with PP3, PP4, PP6, and VC for 4 h, then treated with lead for 24 h. **(A)** The density of Keap1, Nrf2, and TXNIP was determined. **(B)** Represents data on the change in Keap1 protein expression relative to β-actin in different groups; **(C)** represents data on the change in Nrf2 protein expression relative to β-actin in different groups; **(D)** represents data on the change in TXNIP protein expression relative to β-actin in different groups. The valued of the bars indicates the means ± SD (*n* = at least 3). Different superscript letters indicate differences (*P* < 0.05).

The western blotting results for Keap1, Nrf2, and TXNIP are shown in Figure 5A. As shown in Figure 5B, lead exposure significantly increased the expression of Keap1. Surprisingly, all pea peptides and VC significantly decreased the expression of Keap1 compared to the lead group. Among them, PP4 brought its level back to normal and was better than PP3, PP6, and VC (*P* < 0.05). As shown in Figure 5C, lead exposure significantly decreased the expression of Nrf2. Moreover, all pea peptides and VC significantly increased the decrease caused by lead exposure and returned to normal levels; the effect of PP3 and PP4 was better than PP6 and VC (*P* < 0.05). As shown in Figure 5D, lead exposure significantly increased the expression of TXNIP (*P* < 0.05). However, all pea peptides and VC significantly reduced the elevated levels of TXNIP due to lead exposure; among them, only PP6 returned to normal levels, and its effect was better than that of PP3, PP4, and VC (*P* < 0.05).

Many studies report the use of various biological materials like rosemary, ascorbic acid, melatonin and fenobutramide for reducing oxidative stress. Many studies report the use of various biological materials like rosemary, ascorbic acid, melatonin, and fenobutramide for reducing oxidative stress. Evidence from Lv et al’s study showed that lead exposure increases the protein and mRNA levels of Keap1 in HepG2 cells (38). Use of rosemary and ascorbic acid significantly decreased the protein and mRNA levels of Keap1 in lead-exposed cells and protected them from oxidative stress damage, probably because of the regulatory role of the Nrf2-Keap1 antioxidant pathway (38). It has been found that a specific dose of melatonin can significantly enhance the expression of Nrf2, thus alleviating the oxidative stress damage induced by ethanol in BV2 cells during development (39). The antitumor effect of fenobutramide was reported to upregulate TXNIP levels by exacerbating oxidative stress (10). It has also been shown that oxidative stress and inflammasome activation are linked through thioredoxin-interacting proteins (40). Our study illustrates that pea peptides enhance the antioxidant capacity by increasing Nrf2 expression and decreasing Keap1 expression, effectively blocking TXNIP expression and binding to TRX.

## Conclusion

Our results indicate that lead exposure could significantly reduce the viability of PC12 cells, increase ROS and MDA level, decrease the activity of SOD, CAT, GPx, GR, and GSH/GSSG, up-regulate the protein of Keap1 and TXNIP and down-regulated Nrf2 protein expression in PC12 cells. Pea peptides could alleviate lead-induced oxidative stress damage in PC12 cells, and Keap1, Nrf2 and TXNIP play an essential role. The pea peptides could protect PC12 cells from lead-induced oxidative stress damage through the Keap1/Nrf2/TXNIP signaling pathway. Our study would provide a new perspective for the in-depth research of lead neurotoxicity.

## Data availability statement

The original contributions presented in this study are included in the article/supplementary material, further inquiries can be directed to the corresponding author.

## Author contributions

NL: conceptualization, methodology, writing – original draft, funding acquisition, supervision, and project administration. LW: formal analysis, investigation, data curation, writing – review and editing, validation, and visualization. FW: conceptualization, software, funding acquisition, supervision, project administration, and resources. TL: supervision, investigation, and resources. HZ: visualization and data curation. TW: supervision and visualization. MQ: supervision, investigation, and data curation. XH and LS: supervision and funding acquisition. EB: supervision and software. ML: supervision and resources. All authors contributed to the article and approved the submitted version.
